# Supplementary material for: Long-Term Outcomes of Contemporary Percutaneous Coronary Intervention with the Xience Drug-Eluting Stent: Results from a Multicentre Australian Registry
Source: J Clin Med. 2022 Dec 29;12(1):280. doi: 10.3390/jcm12010280 (PMC9821001; doi:10.3390/jcm12010280)
Supplement: Supplementary file 1 [file jcm-12-00280-s001.zip › jcm-2080879-supplementary.pdf]

Supplementary Materials

Table S1. Baseline Clinical Characteristics by Acuity.

Characteristics	Elective			Acute		
n (%)	**BMS 182**	**Xience 560**	***p*-Value**	**BMS 318**	**Xience 429**	***p*-Value**
Age, years, mean (SD)	72.2 (9.7)	67.4 (9.4)	<0.001	71.1 (11.9)	66.2 (10.9)	<0.001
Male	144 (79.1%)	438 (78.2%)	0.80	238 (74.8%)	324 (75.5%)	0.83
Diabetes	45 (24.9%)	147 (26.3%)	0.69	62 (19.6%)	109 (25.4%)	0.063
Hypertension	140 (76.9%)	419 (75.0%)	0.59	229 (72.5%)	298 (70.1%)	0.48
Hypercholesterolaemia	149 (84.7%)	479 (88.2%)	0.22	232 (76.3%)	340 (82.1%)	0.056
Family History of CAD	48 (28.7%)	203 (40.8%)	0.006	89 (29.7%)	169 (43.7%)	<0.001
Smoking (past or current)	102 (57.0%)	285 (52.2%)	0.27	173 (55.3%)	254 (60.2%)	0.18
BMI, kg/m^2 ±SD	29.2 (5.1)	29.1 (4.9)	0.90	28.8 (5.0)	28.8 (4.8)	0.96
ʻLVEF, mean ± SD	57.6 (10.5)	58.7 (9.8)	0.24	53.8 (11.4)	57.0 (9.5)	<0.001
Previous MI	31 (17.0%)	130 (23.3%)	0.075	52 (16.4%)	94 (22.0%)	0.054
Previous Peripheral Vascular Disease	23 (12.6%)	32 (5.7%)	0.002	27 (8.5%)	36 (8.4%)	0.98
Previous PCI	35 (19.3%)	211 (37.7%)	<0.001	50 (15.8%)	127 (29.6%)	<0.001
Previous Cerebrovascular disease	20 (11.0%)	33 (5.9%)	0.022	31 (9.7%)	28 (6.6%)	0.11
Previous CABG	24 (13.3%)	55 (9.8%)	0.20	25 (7.9%)	43 (10.0%)	0.31
Renal impairment	14 (8.3%)	18 (3.6%)	0.014	24 (7.7%)	23 (5.7%)	0.28
**Clinical presentation**						<0.001
STEMI				100 (31.4%)	43 (10.0%)	
NSTEMI				145 (45.6%)	215 (50.1%)	
Unstable angina				73 (23.0%)	171 (39.9%)	
Elective	182 (100.0%)	560 (100.0%)				
Cardiogenic Shock	0 (0.0%)	1 (0.2%)	0.57	5 (1.6%)	1 (0.2%)	0.043

CAD-coronary artery disease; BMI-Body mass index; PCI- Percutaneous coronary intervention; CABG-Coronary artery bypass grafting; MI-Myocardial infarction; STEMI-ST-elevation myocardial infarction; NSTEMI-Non-ST-elevated myocardial infarction; SD-Standard deviation.

Table S2. Lesion & procedural characteristics by Acuity.

	Elective			Acute		
	**BMS 182**	**Xience 560**	***p***	**BMS 318**	**Xience 429**	***p***
Lesions/procedure, mean (SD)	1.2 (0.5) (*n* = 182)	1.4 (0.7) (*n* = 560)	<0.001	1.2 (0.5) (*n* = 318)	1.4 (0.6) (*n* = 429)	0.002
Access site			0.035			<0.001
Radial				0 (0.0%)	3 (0.7%)	
Femoral	48 (26.4%)	195 (34.8%)		69 (21.7%)	142 (33.2%)	
Brachial	134 (73.6%)	365 (65.2%)		249 (78.3%)	283 (66.1%)	
Lesion Type			0.089			0.001
De novo	176 (96.7%)	531 (94.8%)		312 (98.1%)	396 (92.3%)	
Restenosis	0 (0.0%)	2 (0.4%)				

In-stent restenosis	3 (1.6%)	25 (4.5%)		4 (1.3%)	29 (6.8%)	
Other	2 (1.1%)	2 (0.4%)		2 (0.6%)	4 (0.9%)	
Unknown	1 (0.5%)	0 (0.0%)				
ACC/AHA Morphology			<0.001			0.048
A	32 (17.7%)	45 (8.2%)		38 (12.0%)	41 (9.6%)	
B1	82 (45.3%)	234 (42.5%)		147 (46.4%)	170 (39.7%)	
B2 or C	67 (37.0%)	271 (49.3%)		132 (41.6%)	217 (50.7%)	
Target vessel			0.010			0.001
RCA	70 (38.7%)	162 (29.3%)		132 (41.6%)	129 (30.1%)	
LMCA	3 (1.7%)	7 (1.3%)		2 (0.6%)	2 (0.5%)	
LAD	63 (34.8%)	270 (48.9%)		98 (30.9%)	193 (45.1%)	
LCx	35 (19.3%)	98 (17.8%)		70 (22.1%)	91 (21.3%)	
Bypass Graft	10 (5.5%)	15 (2.7%)		15 (4.7%)	13 (3.0%)	
Chronic total occlusion	1 (0.6%)	15 (2.7%)	0.083	4 (1.3%)	7 (1.6%)	0.67
Multi-vessel disease	59 (32.4%)	195 (34.8%)	0.60	111 (34.9%)	154 (35.9%)	0.92
Bifurcation lesion	12 (6.7%)	71 (12.9%)	0.023	12 (3.8%)	42 (9.8%)	0.002
Lesion success	181 (100.0%)	548 (99.5%)	0.32	315 (99.4%)	426 (99.5%)	0.76
Stents / procedure, mean (SD)	1.3 (0.7) (n = 182)	1.5 (0.8) (n = 560)	0.029	1.3 (0.7) (n = 318)	1.5 (0.8) (n = 429)	0.005
Stent length, mean (SD)	16.4 (4.4) (n = 182)	18.9 (6.1) (n = 559)	<0.001	17.4 (4.9) (n = 317)	18.4 (6.1) (n = 429)	0.020
Stent length >20 mm	24 (13.2%)	178 (31.8%)	<0.001	61 (19.2%)	121 (28.2%)	0.005
Stent diameter, mean (SD)	3.4 (0.7) (n = 182)	2.9 (0.4) (n = 559)	<0.001	3.3 (0.6) (n = 317)	2.9 (0.5) (n = 429)	<0.001
Vessel ≤2.5 mm	22 (12.1%)	135 (24.1%)	<0.001	42 (13.2%)	112 (26.1%)	<0.001
IIb/IIIa use during procedure	9 (4.9%)	18 (3.2%)	0.28	38 (11.9%)	37 (8.6%)	0.13

RCA-Right coronary artery; LMCA-Left main coronary artery; LAD-Left anterior descending artery; LCX-Left circumflex artery; BMS-Bare metal stent; SD-Standard deviation.
